# Attitudes to aging are associated with cognition and biomarker status in older adults at risk for Alzheimer's disease

**DOI:** 10.1002/alz70858_106275

**Published:** 2025-12-25

**Authors:** Franciska Otaner, Caitlin S. Walker, Adrián Noriega de la Colina, Linda Li, Carolynn Boulanger, Nagashree Thovinakere, Alix Noly‐Gandon, Garance Barnoin, Mitchell Bennett, Jillian Caplan, Laurence Côté, Sarah Elbaz, Shania Fock Ka Bao, Ryan Kara, Nicolas Lavoie, Maggie Nguyen, Helen Pallett‐Wiesel, Johanie Victoria Piché, Andreanne Powers, Sofia Ricciardelli, Kayla Williams, Christine Dery, Jennifer Tremblay‐Mercier, Judes Poirier, Sylvia Villeneuve, Arthur F. Kramer, Maiya R. Geddes, PREVENT‐AD Research Group

**Affiliations:** ^1^ Faculty of Medicine and Health Sciences, McGill University, Montreal, QC, Canada; ^2^ Montreal Neurological Institute‐Hospital Cognitive Neuroscience Research Group, McGill University, Montreal, QC, Canada; ^3^ The Neuro, Faculty of Medicine, McGill University, Montreal, QC, Canada; ^4^ Centre for Studies in the Prevention of Alzheimer's Disease, Douglas Mental Health Institute, McGill University, Montreal, QC, Canada; ^5^ Massachusetts Institute of Technology, Cambridge, MA, USA; ^6^ McGill University Research Centre for Studies in Aging, McGill University, Montreal, QC, Canada; ^7^ Department of Pharmacology and Therapeutics, McGill University, Montreal, QC, Canada; ^8^ Rotman Research Institute, University of Toronto, Toronto, ON, Canada; ^9^ McGill University, Montreal, QC, Canada; ^10^ Faculty of Medicine, Laval University, Quebec City, QC, Canada; ^11^ Faculty of Pharmacy, Université de Montréal, Montreal, QC, Canada; ^12^ School of Physical and Occupational Therapy, Faculty of Medicine and Health Sciences, McGill University, Montreal, QC, Canada; ^13^ Douglas Mental Health University Institute, Montreal, QC, Canada; ^14^ McConnell Brain Imaging Centre (BIC), MNI, Faculty of Medicine, McGill University, Montreal, QC, Canada; ^15^ Beckman Institute, University of Illinois, Urbana, IL, USA; ^16^ Center for Cognitive & Brain Health, Northeastern University, Boston, MA, USA

## Abstract

**Background:**

Attitudes toward aging influence longevity as well as physical and psychological health, yet their relationship with cognition and Alzheimer's disease (AD) remains largely unknown. We hypothesized that positive attitudes toward aging would predict better cognition and diminished risk on AD blood‐based biomarker profiles in older adults at risk for AD. Understanding the influence of subjective outlook on AD risk and cognitive resilience could provide valuable insights into disease mechanisms.

**Method:**

A subsample of older adults (*n* = 54; 39 women) with a family history of AD from the PREVENT‐AD longitudinal cohort participated in this study. Participants completed the Attitudes to Ageing Questionnaire (AAQ‐24), which provided three scores: psychosocial loss, psychological growth, and physical change. Cognitive assessments included episodic memory (Rey Auditory Verbal Learning Test, RAVLT) and executive functions (Delis‐Kaplan Executive Function System‐Color Word Interference Test, D‐KEFS‐CWIT). The AD blood‐based biomarkers *p*‐tau217 and Aβ42/40 ratios were also measured. A Weighted Least Squares regression model was used to correct for heteroscedasticity and to test whether attitudes toward aging predicted cognitive performance and plasma biomarker profiles. All analyses were corrected for multiple comparisons using the Bonferroni method, with covariates of non‐interest, including age, sex, education, depression, and *APOE4* carriership status, incorporated in the model.

**Result:**

Positive attitudes to aging were associated with better recall and recognition (RAVLT) and improved executive function including switching and inhibitory control (D‐KEFS‐CWIT) (*p* <0.00077, Bonferroni corrected), while negative attitudes showed the opposite pattern. In addition, negative attitudes to aging were associated with a lower Aβ42/40 ratio, while positive attitudes were linked to lower *p*‐tau217 (*p* <0.0167, Bonferroni corrected).

**Conclusion:**

These findings indicate that positive attitudes toward aging are significantly linked to both enhanced cognitive performance and a lower risk profile in AD blood‐based biomarkers. Our results suggests that life outlook could either be an early indicator of disease or, alternatively, a modifiable AD risk factor.

